# Metabolic niches in the rhizosphere microbiome: dependence on soil horizons, root traits and climate variables in forest ecosystems

**DOI:** 10.3389/fpls.2024.1344205

**Published:** 2024-04-05

**Authors:** Pulak Maitra, Katarzyna Hrynkiewicz, Agnieszka Szuba, Andrzej M. Jagodziński, Jubair Al-Rashid, Dipa Mandal, Joanna Mucha

**Affiliations:** ^1^ Institute of Dendrology, Polish Academy of Sciences, Kórnik, Poland; ^2^ Department of Microbiology, Faculty of Biological and Veterinary Sciences, Nicolaus Copernicus University, Toruń, Poland; ^3^ Department of Game Management and Forest Protection, Faculty of Forestry and Wood Technology, Poznań University of Life Sciences, Poznań, Poland; ^4^ Tianjin Institute of Industrial Biotechnology, University of Chinese Academy of Sciences, Tianjin, China; ^5^ Institute of Microbiology, University of Chinese Academy of Sciences, Beijing, China; ^6^ Department of Forest Entomology and Pathology, Faculty of Forestry and Wood Technology, Poznań University of Life Sciences, Poznań, Poland

**Keywords:** soil microbes, root exudation, soil horizon, root traits, drought, temperature

## Abstract

Understanding belowground plant-microbial interactions is important for biodiversity maintenance, community assembly and ecosystem functioning of forest ecosystems. Consequently, a large number of studies were conducted on root and microbial interactions, especially in the context of precipitation and temperature gradients under global climate change scenarios. Forests ecosystems have high biodiversity of plants and associated microbes, and contribute to major primary productivity of terrestrial ecosystems. However, the impact of root metabolites/exudates and root traits on soil microbial functional groups along these climate gradients is poorly described in these forest ecosystems. The plant root system exhibits differentiated exudation profiles and considerable trait plasticity in terms of root morphological/phenotypic traits, which can cause shifts in microbial abundance and diversity. The root metabolites composed of primary and secondary metabolites and volatile organic compounds that have diverse roles in appealing to and preventing distinct microbial strains, thus benefit plant fitness and growth, and tolerance to abiotic stresses such as drought. Climatic factors significantly alter the quantity and quality of metabolites that forest trees secrete into the soil. Thus, the heterogeneities in the rhizosphere due to different climate drivers generate ecological niches for various microbial assemblages to foster beneficial rhizospheric interactions in the forest ecosystems. However, the root exudations and microbial diversity in forest trees vary across different soil layers due to alterations in root system architecture, soil moisture, temperature, and nutrient stoichiometry. Changes in root system architecture or traits, e.g. root tissue density (RTD), specific root length (SRL), and specific root area (SRA), impact the root exudation profile and amount released into the soil and thus influence the abundance and diversity of different functional guilds of microbes. Here, we review the current knowledge about root morphological and functional (root exudation) trait changes that affect microbial interactions along drought and temperature gradients. This review aims to clarify how forest trees adapt to challenging environments by leveraging their root traits to interact beneficially with microbes. Understanding these strategies is vital for comprehending plant adaptation under global climate change, with significant implications for future research in plant biodiversity conservation, particularly within forest ecosystems.

## 1 Introduction

Forest ecosystems, which cover one-third of the world’s land area and encompass more than 24.6 million square kilometers, are crucial for global ecosystem services and carbon cycling, particularly under the influence of climate change (Viet et al., 2022). These ecosystems, predominantly temperate and boreal forests, consist of diverse deciduous and coniferous trees that are well-adapted to a broad range of environmental conditions, especially in terms of temperature and precipitation (Tasenkevich et al., 2023). The soils of these forests support a rich array of prokaryotic and eukaryotic microbial communities, including bacteria and fungi. These organisms are essential for maintaining ecosystem health and are key players in ecosystem carbon and nutrient cycling (Bardgett et al., 2014a; Baldrian, 2017). Some microbes are particularly adept at decomposing refractory soil organic matter (SOM) and cellulose, a capability linked to their filamentous growth and ability to excrete extracellular enzymes (Bodeker et al., 2014; Gómez et al., 2020). Furthermore, these forest soils are home to diverse communities of saprotrophic and mycorrhizal fungi, integral to the ecosystem’s functioning and heavily reliant on the interactions within the belowground rhizosphere (Voříšková et al., 2014). Understanding these complex microbial interactions and the dynamics of below-ground carbon allocation via root exudation is vital for predicting carbon balance in terrestrial ecosystems, especially in the face of ongoing global changes (DeAngelis et al., 2015).

**Figure 1:**
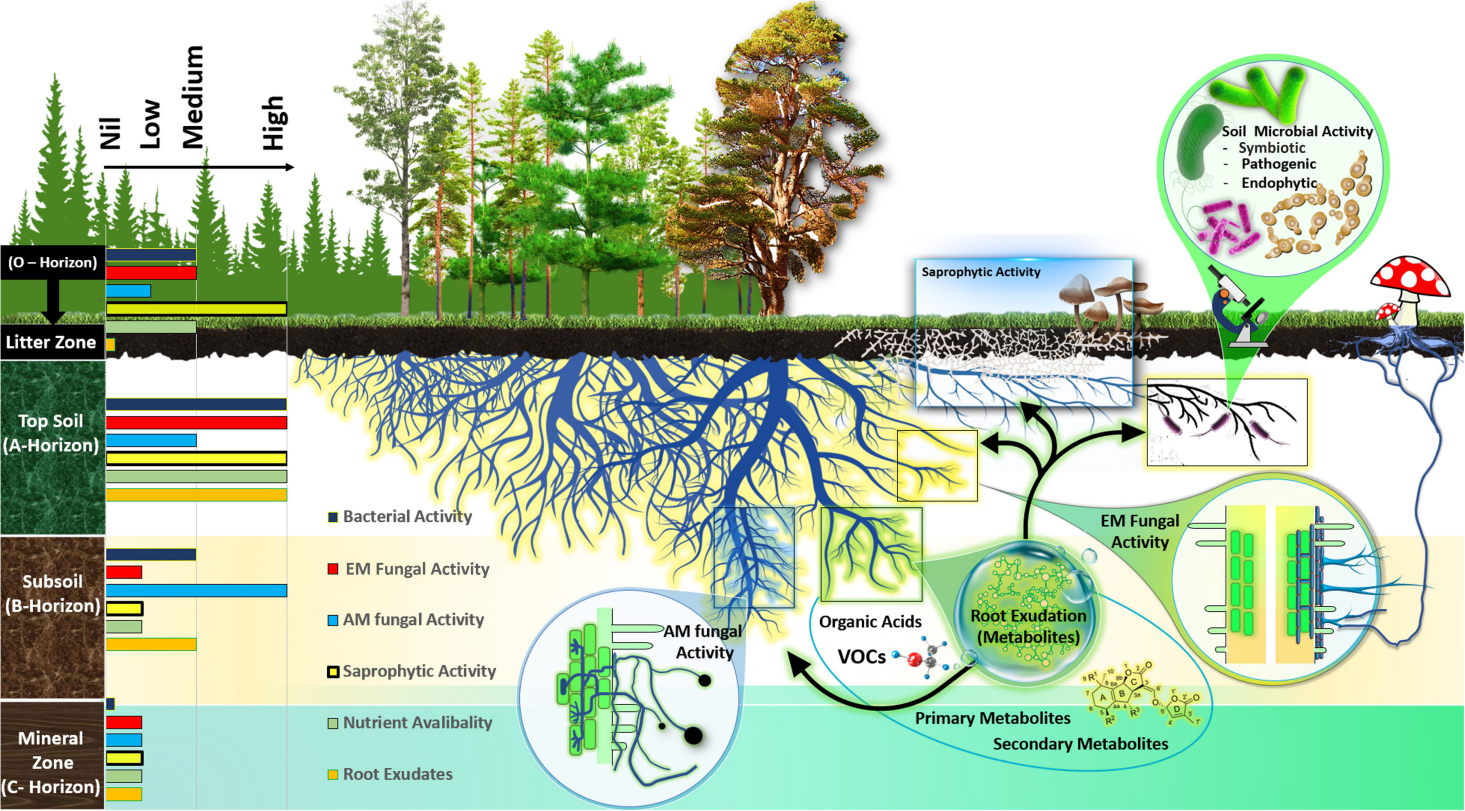
General overview of the metabolic niche of the rhizosphere microbial functional group in forest ecosystems. Different soil horizons differ in the activity of ectomycorrhizal fungi (ECM), arbuscular mycorrhizal fungi (AM), saprotrophic fungi, and bacterial communities as well as exudation of root metabolites and nutrient availability, demonstrated by currently available research.

Fine roots of forest trees, highly active metabolic zones within the soil, are key in the interactions with subterranean microorganisms (**Figure 1**) (King et al., 2023). The communication between plants and soil microbes is predominantly mediated through chemical signals, as indicated by Pascale et al. (2020) and Williams and de Vries (2020). Root systems of forest trees excrete various chemical compounds, collectively known as metabolites, which are essential for diverse biological processes (**Figure 1**). These metabolites encompass primary metabolites such as amino acids, organic acids, and sugars, along with secondary metabolites like phenolic chemicals, and volatile compounds including terpenoids and sulfides. Such root exudations of forest trees have a significant impact on numerous ecosystem functions. For example, they affect soil microbial dynamics (Jing et al., 2023), enhance plant resilience to non-living stress factors (Williams and de Vries, 2020; Jiang et al., 2023), and contribute to nutrient cycling and the stability of soil structure (Mommer et al., 2016; Wang et al., 2021a; Wang et al., 2021b). Additionally, roots release various other metabolites, accounting for 10–50% of the carbon fixed by plants (Nguyen, 2003; Massalha et al., 2017; Prescott and Grayston, 2023), thereby playing an integral role in the global carbon cycle.

Microbial diversity and abundance in the rhizosphere are affected by plant developmental stage, genotypes, and soil parameters (Ofek-Lalzar et al., 2014; Schlaeppi et al., 2014; Bulgarelli et al., 2015; Paredes and Lebeis, 2016). For instance, the main soil parameters, e.g. changing soil organic matter distribution with soil depth, influence root exudation as well as microbial diversity and functioning in temperate forest ecosystems (Tückmantel et al., 2017; Prada-Salcedo et al., 2022) (**Figure 1**). However, this assemblage of microbes in different soil horizons may change due to the changes in root morphology and exudation of forest trees along a wide range of environmental gradients, for example drought or temperature (Meier et al., 2020; Veach et al., 2020). It has even been hypothesized that plants dynamically recruit soil microbes by secreting metabolites in the rhizosphere that ideally stimulate rhizosphere microorganisms or endophytic fungi and bacteria that are advantageous to plant growth by helping them cope with abiotic stresses (Sasse et al., 2018; Hildebrand et al., 2023). Knowledge about the responses of root metabolites and microbial assemblages in the roots and rhizosphere in different horizons of forest soil along environmental gradients of drought and temperature will increase our aptitude to forecast the global change effects on soil organic matter decomposition and C cycling (Steidinger et al., 2019). Therefore, a better understanding of rhizosphere microbial interactions and below ground C allocation via root exudation is important for predicting C balance in the terrestrial ecosystem under global change scenarios.

In this review, we address the existing knowledge about the metabolic niche of rhizosphere microbes and questions which were previously unanswered. How microbial activity differ in different soil horizon of forest ecosystems? How do root traits and exudation change within soil profiles? What are the impacts of different metabolites on microbial functional groups? How is metabolite change linked to root traits to influence microbial functional groups in forest soil? And finally, how do climate factors influence root traits, root exudation and microbiome dynamics in forest ecosystem? The goal of this review is to answer these questions for forest ecosystems to summarize the current information about the interactions among root traits, rhizosphere metabolites and microbial communities, and about their response to climate change, which have particular implications for future research investigations into the microbial ecology of forest soils.

## 2 Microbial activity differs in different soil horizons

Soil horizons of forest areas are distinguished by their unique soil properties, exhibiting varied physical, chemical, and microbiological characteristics (Rumpel and Kögel-Knabner, 2011; Horodecki and Jagodziński, 2017; Horodecki et al., 2019). The O horizon, known as the litter zone, is rich in organic materials and darker in color, comprising decomposing plant materials like leaves, needles, and twigs (Horodecki and Jagodziński, 2017). The A horizon, or topsoil, contains a mix of decomposed plant matter and soil minerals. The C horizon, or mineral horizon, is marked by lower organic matter content, a result of organic matter decomposition (Voříšková et al., 2014; Hartemink et al., 2020). The B horizon represents a blend of mineral zone soil and subsoil components (**Figure 1**). The distribution of soil microbial communities in forest ecosystem varies across these horizons due to differing levels of organic matter decomposition (Jiao et al., 2018; Mundra et al., 2021; Luo et al., 2023). High-throughput sequencing has shown distinct fungal community clusters in different soil horizons (McGuire et al., 2013b). For example, in tropical forests, the O-horizon is predominantly inhabited by saprotrophic fungi, while the A-horizon below it is mainly occupied by ectomycorrhizal fungi (McGuire et al., 2013a). Furthermore, the horizontal separation of ectomycorrhizal and saprotrophic fungi in forest soil layers, reflecting their specific functional roles in decomposing organic matters at various stages of decay (Dickie et al., 2002; O'Brien et al., 2005; Lindahl et al., 2007; McGuire et al., 2013a; Lustenhouwer et al., 2020). For instances, in tropical, temperate and boreal forests reveal that ectomycorrhizal fungi are more abundant in the lower litter zone, while saprotrophs are prevalent in the upper layers (McGuire et al., 2013a; Carteron et al., 2021). This stratification is due to the ability of some ectomycorrhizal fungi to decompose organic matter for nitrogen, while they primarily source carbon from their host plants. On the other hand, saprotrophic fungi derive their carbon from decomposing organic matter and are predominantly found in shallower soil layers that are abundant in carbon-rich substrates (Lindahl et al., 2007; Carteron et al., 2021; Marañón-Jiménez et al., 2021). In pine forests, it has been observed that the overall fungal biomass is considerably more abundant in the litter and humus layers compared to the deeper mineral zone (Adamo et al., 2022). However, in oak forests, the dominant ectomycorrhizal fungi *Cortinarius* and *Sebacina* are more prevalent in the A horizon, while saprotrophic fungi like the genus *Preussia* dominate the litter layer (Adamo et al., 2022). Furthermore, in Beech and Oak forest ecosystems, the ectomycorrhizal genera *Amanita, Melanogaster, Odontia, Sistotrema*, and *Telephora* exhibited higher abundances in the topsoil compared to the subsoil (Frey et al., 2021). Moreover, in temperate forests, ectomycorrhizal fungi from the Boletales order were enriched in the organic layer (litter zone), while Russulales and Cantharellales were predominant in the topsoil (A-horizon) (Khokon et al., 2021). This pattern is attributed to the saprotrophic nature of Boletales, which are predominant in the organic layer, and the symbiotic nature of Russulales in the mineral soil, indicating territorial behavior among members of the these order, as indicated by Pena et al. (2017).

A prevailing model about the spatial distribution of mycorrhizal types proposes that, in environments where they coexist, ectomycorrhizal (EcM) fungi are typically more abundant in organic soil layers and in topsoil, whereas arbuscular mycorrhizal (AM) fungi are more commonly found in mineral-rich sub soil horizons (Smith and Read, 2008). For instance, in soil ecosystems, the dominance of ectomycorrhizal fungi is more pronounced near the surface, while arbuscular mycorrhizal (AM) fungi are more abundant in deeper layers, showing a decrease in ectomycorrhizal compared to AM colonization as soil depth increases in northern hardwood forests (Nash et al., 2022). AM fungi’s enhanced ability to absorb phosphorus (P), especially inorganic P (Shen et al., 2020), compared to nitrogen (N), and the proficiency of ectomycorrhizal fungi in acquiring N (Bicharanloo et al., 2021), suggest that each group thrives in soil layers where their preferred nutrient is scarce (Read, 1991). This perspective is reinforced by research that highlights varying root colonization patterns in temperate and rain forest ecosystems where different types of mycorrhizae coexist (Moyersoen et al., 1998) and the distinct acquisition strategies (Phillips et al., 2013). This distribution pattern is exemplified in case of *Populus tremuloides* growing in boreal forests, where roots in the upper soil are predominantly colonized by ectomycorrhizal fungi, whereas deeper roots are mainly colonized by AM fungi (Neville et al., 2002). Moreover, the competitive dynamics between ectomycorrhizal and saprotrophic fungi influence their soil depth distribution. For instances, in temperate and boreal forests, competition for soil resources has been suggested to confine saprotrophic fungi primarily to the uppermost organic soil layers in areas dominated by ectomycorrhizal trees. Conversely, in AM fungi-dominated forests, a lower overlap in ecological niches allows saprotrophic fungi to extend their dominance to deeper soil layers (Carteron et al., 2021).

The structure of bacterial communities in soil, including the composition and abundance of different taxa, changes with soil depth, tending to be more diverse in the topsoil. This diversity and abundance is correlated with the soil’s physical properties and the availability of resources across soil horizons in different forest ecosystems (Eilers et al., 2012; Chu et al., 2016; Shao et al., 2019). These shifts in community structure are marked by changes in the relative abundance of dominant bacterial groups. For example, in a temperate forest ecosystem, there are notably high populations of Actinobacteria, Bacteroidetes, and Proteobacteria in topsoil areas that have a high concentration of soil organic matter (SOM) (Shao et al., 2019). This distribution pattern is similar to what is found in grassland and agricultural soils and is linked to variations in soil nutrients or SOM levels (Will et al., 2010; Sagova-Mareckova et al., 2016). Our analysis of the above literature about the vertical distribution of microbial communities allowed us to classify abundance and activity of bacterial and fungal communities as high, medium, and low across the forest soil horizons. This analysis is summarized in the left section of **Figure 1**. However, studies focusing on the characterization of bacterial functional group across different forest soil horizons, particularly mutualistic group, have been comparatively limited.

## 3 Impact of different metabolites on microbial functional groups

**Figure 2:**
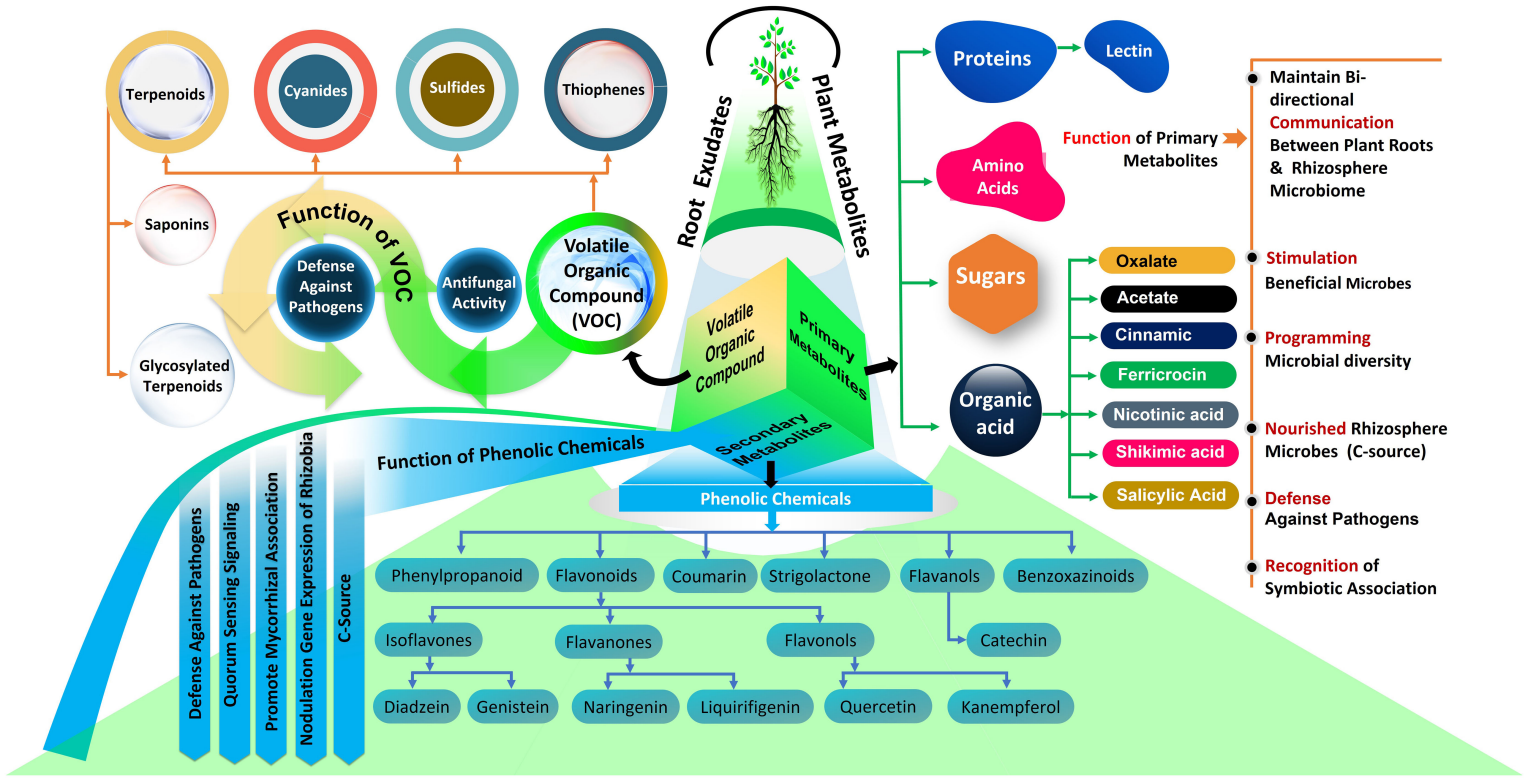
Functions of root metabolites in soil microbial activity.

Recent research has increasingly highlighted the crucial role that root exudates play in the interactions between forest trees and microbes in the rhizosphere (Sasse et al., 2018; Pascale et al., 2020; Williams and de Vries, 2020; Oppenheimer-Shaanan et al., 2022; Jing et al., 2023; Staszel-Szlachta et al., 2024). In this context, it is well understood that plants supply photosynthetic carbon to rhizospheric microbes and release various key molecules (**Figure 2**) into the soil, thereby fostering a bidirectional relationship. In this interaction, microbes also contribute to plant growth by aiding in nutrient acquisition and enhancing tolerance to abiotic and biotic stress. These rhizosphere metabolites play diverse roles influencing various fungal and bacterial functional groups as well as their functions in belowground ecosystems as summarized below (**Figure 2**).

### 3.1 Root metabolites influence on fungal functional groups

Root metabolites in the rhizosphere soil have a wide range of potential for attracting and repelling distinct fungal strains and hence affect the rhizosphere fungal population (Chagas et al., 2018). Plants influence the enrolment of mycorrhizal fungi in the rhizosphere through the release of specific chemical signals into the soil (Korenblum et al., 2022). Studies have also shown that differences in rhizosphere communities between different tree species are the result of selective attraction of specific fungi through plant root secretions (Prescott and Grayston, 2013; Jing et al., 2023). For instances, ectomycorrhizal associations of forest trees typically lead to an increase in the exudation of organic acids, or a change in the types of organic acids released by roots (Johansson et al., 2008, 2009; Jourand et al., 2014; Abdulsalam et al., 2021). Furthermore, van Hees et al. (2006) studied *Hebeloma* ectomycorrhizal species in symbiosis with *Pinus sylvestris* (Scots pine) and discovered that these mycorrhizal associations led to the secretion of specific compounds. These included oxalate and ferricrocin, along with trace amounts of malonate and acetate, which were distinctly present in soils around mycorrhizal Scots pine but absent in soils surrounding non-mycorrhizal Scots pine. Similarly, a study on oak roots colonized by the ECM fungus *Tuber indicum* showed an accumulation of carbohydrates and organic acids in the ectomycorrhizal root tips (Li et al., 2018). Additionally, carbon produced by trees in the form of root exudates is rapidly transferred to mycorrhizal partners via mycelial networks in the soil (Kaiser et al., 2015; Gorzelak et al., 2020; Hawkins et al., 2023). Moreover, it’s hypothesized that fungal hyphae release labile carbon, such as carbohydrates and amino acids, into the soil away from the roots, potentially enhancing the decomposition of organic matter by free-living soil saprotrophs (Frey, 2019). While the knowledge of the specific functions of the specific root metabolites in relation to fungal functional groups in forest ecosystems is limited, the information presented below is based on studies conducted *in vitro* and in agricultural ecosystems have demonstrated their distinct roles. For instance, certain root exudates, such as strigolactone, have been found to trigger the activation of AM fungal genes related to the initiation of hyphal growth near plant roots (Martin et al., 2008; Clark and Bennett, 2023). Strigolactones enhance the hyphal branching of AM fungi, which is a crucial step in the colonization process of plant roots. This not only aids in the establishment of the symbiosis but also in the fungal acquisition of nutrients, particularly phosphorus, from the soil, which are then transferred to the plant, enhancing its nutrient uptake and stress tolerance. Studies by Akiyama and Hayashi (2006), for example, have demonstrated that the application of synthetic strigolactones can significantly increase the colonization of plant roots by AM fungi, underlining the direct impact of these metabolites on mycorrhizal fungi. Furthermore, symbiotic relationships between non-legumes and AM fungi involve root exudate compounds such as strigolactone 5-deoxystrigol (Yoneyama, 2019), sugars (Fang and St. Leger, 2010), and carbohydrates (Kiers et al., 2011). Moreover, plants that effectively battle root fungal pathogen invasion frequently produce unique volatile organic compounds that perform direct defensive functions (Delory et al., 2016). Plant roots secrete triterpenoids, which are among the most powerful antifungal defensive chemicals (Zhong et al., 2022). More precisely, saponins, glycosylated triterpenoids released by cereals, denote well recognized constitutive defense substances against fungal pathogens, as has been proved for the antifungal properties of avenacin secreted by oat roots (Osbourn, 2003). These studies reveal how different root metabolites can selectively influence fungal groups, offering insights that might be applicable to forest ecosystems. However, research specifically for forest trees in this area is still limited or yet to be described. Therefore, it remains to be verified whether these findings are compatible with forest ecosystems.

### 3.2 Root metabolites influence bacterial functional groups

The dynamic profile of metabolites at the root-soil interface correlates with microbial substrate preferences, which can often be predicted from genomic sequences. Different populations of forest trees secrete a variety of metabolites that have unique effects on the composition of bacterial communities. For instance, untargeted metabolite studies of *Pinus tabulaeformis* plants have shown that functional carbon metabolites, such as hippuric acid, cytidine-5´-monophosphate, lactic acid, glucose, and spermidine, influence the microbial populations in rhizosphere soil, including Actinobacteria, Acidobacteria, Proteobacteria under nitrogen addition in a subtropical forest ecosystems (Jing et al., 2023). Furthermore, previous investigation demonstrated that addition of sugars from pine root exudates to soil influences the abundance and activity of various bacterial taxa, like Proteobacteria, Actinobacteria, and Firmicutes (Shi et al., 2011). Moreover, *Pinus sylvestris* releases a variety of rhizosphere metabolites, such as fatty acids, monoacylglycerides, and diterpenes, in a temperate forest; these metabolites significantly affect the diversity and composition of rhizosphere bacterial communities (Bi et al., 2022). Moreover, sugars and organic acid released as root exudates influenced the diversity and community assembly of free-living N_2_ fixing diazotrophic communities in forest soil (Ding et al., 2021). For examples, in a desert forest ecosystem, the presence of key diazotroph species like *Azotobacter, Azospirillum, Bradyrhizobium, and Mesorhizobium* is significantly correlated with the levels of various sugars and organic acid exudates (Ding et al., 2021). However, research into how root exudates affect the soil bacteria and their functional groups (decomposers, mutualists, pathogens, and lithotrophs) is less extensive in forest ecosystems. Many studies conducted *in vitro* with model plants and in agricultural settings, as detailed in the information presented below, have demonstrated the specific roles of metabolites in fostering associations with certain bacterial functional groups. For instance, the plant growth-promoting bacteria *Bacillus subtilis* was recruited to the rhizosphere through malic acid in root exudates, particularly after infection by foliar pathogens (Rudrappa et al., 2008; Rekha et al., 2018). Recent findings also indicate that coumarins, a subset of phenolic compounds, display variable toxicity levels against pathogenic bacteria (Stringlis et al., 2018, 2019; Voges et al., 2019). Furthermore, flavonoids and strigolactones, are two additional naturally occurring phenolic metabolites that act as signaling molecules in the formation of well-studied mutualistic associations of plant hosts with *Rhizobium* bacteria (Subramanian et al., 2007; Dong and Song, 2020). Flavonoids have been linked to the control of nodulation genes expression in nitrogen-fixing rhizobia (Cesco et al., 2010; Hassan and Mathesius, 2012) as well as *Pseudomonas aeruginosa* quorum-sensing (QS) signals (Vandeputte et al., 2011). Flavonoids secreted by plant roots are detected by Rhizobium, which in turn produces Nod factors that stimulate root hair curling and nodule formation, leading to the establishment of a symbiotic relationship. This interaction significantly enhances the plant’s ability to fix atmospheric nitrogen. Empirical studies, such as that by Subramanian et al. (2007), have demonstrated that flavonoid-deficient mutants of legumes show reduced nodulation and symbiotic nitrogen fixation, highlighting the direct influence of these metabolites on nitrogen-fixing bacteria. Beyond phenolics, some other root secondary metabolites involved in the balance between roots and bacterial populations have been demonstrated, including benzoxazinoids (Hu et al., 2018; Cotton et al., 2019), triterpenes and camalexin (Huang et al., 2019). In addition, plants also release proteins as root exudates (De-la-Peña et al., 2010). In this context, lectins are among the most investigated proteins which act as defenses against pathogens and recognition factors in symbiotic associations (De Hoff et al., 2009; Rocha et al., 2015; Bellande et al., 2017). However, it’s important to note that while this evidence is compelling in controlled environments and agricultural settings, direct and specific knowledge about the functions of these metabolites towards bacterial functional groups in forest ecosystems is not as extensive. Therefore, while these findings offer valuable perspectives, caution should be exercised in directly applying these insights to forest ecosystems, and there is a need for more focused research in these natural environments to understand the roles of root metabolites and their associations with bacterial functional groups.

## 4 Root morphological traits influence root exudation

**Figure 3:**
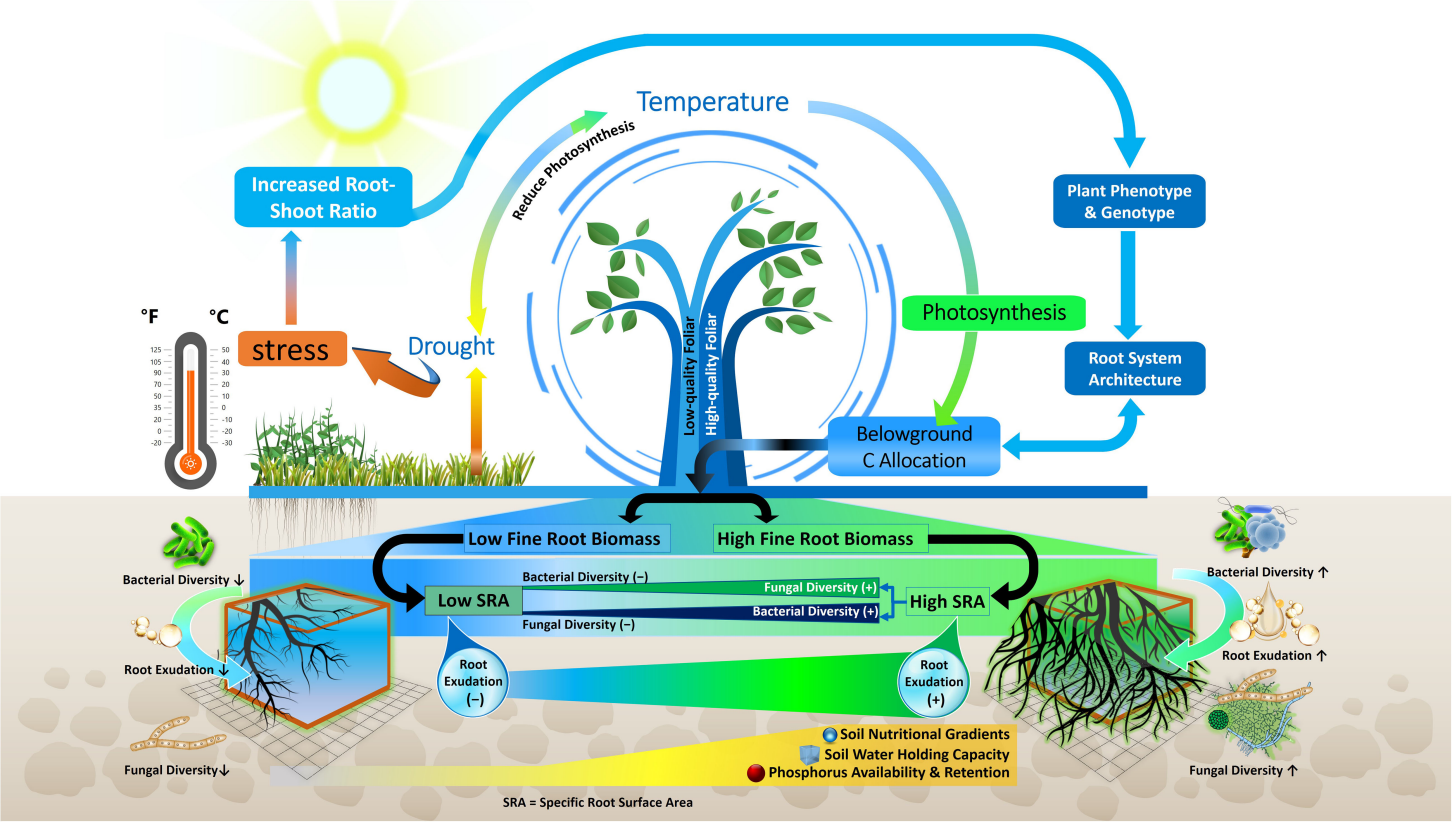
Schematic of the relationships among root traits, climate factors, root exudation and microbiome composition. Drought and temperature affect photosynthetic activity and belowground C allocation which in turn influence fine root biomass and specific root surface area (SRA), root exudation, microbial diversity and nutrient availability. Decreased root exudation (-); increased root exudation (+); higher fungal and bacterial diversity (↑); and Lower fungal and bacterial diversity (↓).

**Figure 4:**
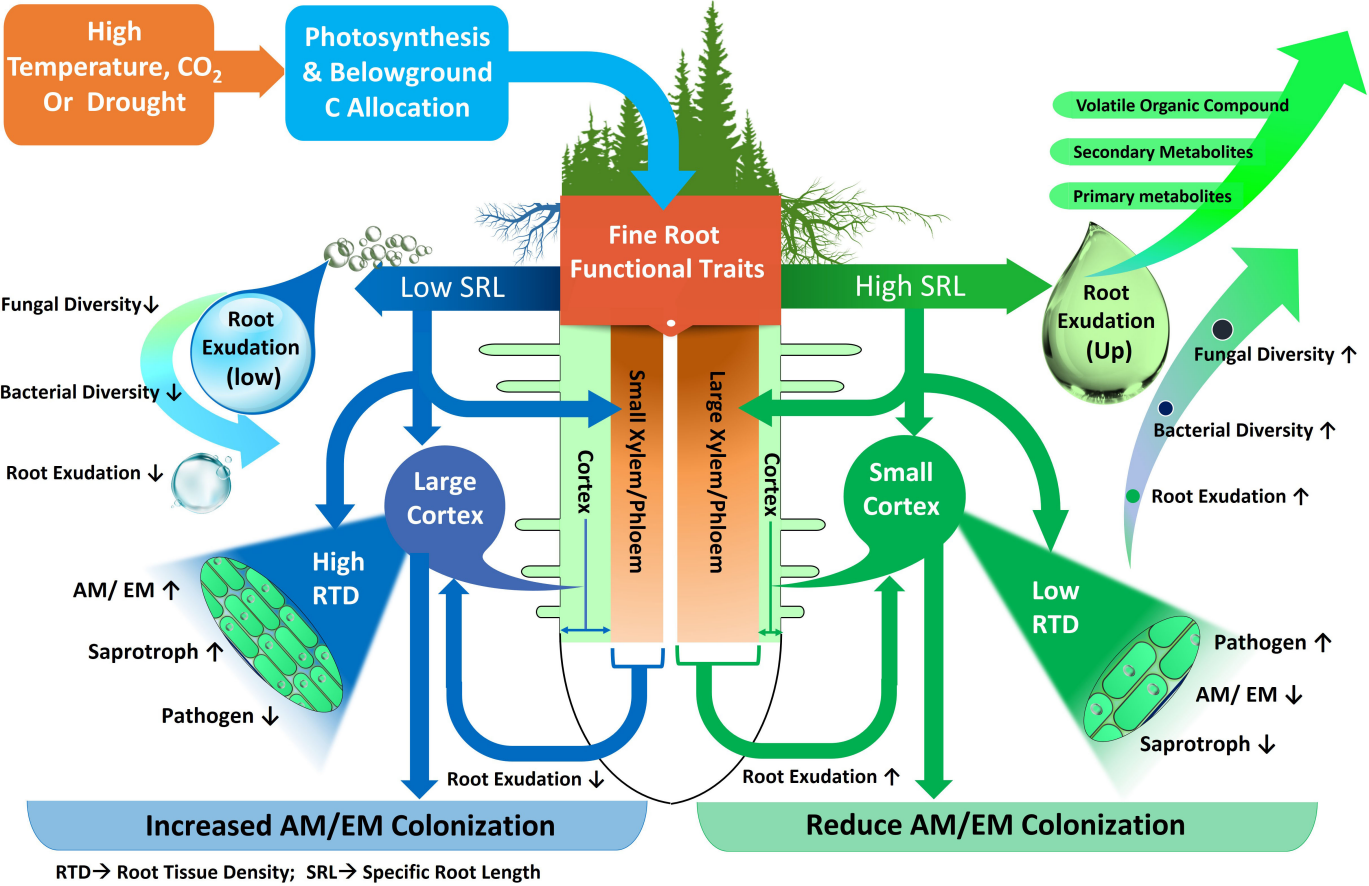
Fine root functional traits and their relationships with environmental variables, root metabolites and groups of microorganisms. Precipitation and temperature affect fine root functional traits with low or high specific root length (SRL). Roots with low SRL possess small Xylem-Phloem and large cortex with high root tissue density (RTD). Roots with high SRL possess big Xylem-Phloem and small cortex with low root tissue density (RTD). Decreased (↓) or increased (↑) fungi, bacteria, ectomycorrhiza (EM), arbuscular mycorrhiza (AM), saprotroph and pathogen diversity and activity.

Environmental changes lead to a variety of phenotypic and metabolic alterations in forest tree roots, which in turn impact the profile of soil metabolites (Karst et al., 2017; Gargallo-Garriga et al., 2018; Hartman and Tringe, 2019). In previous investigations, the root morphological traits that influence rhizosphere metabolites in temperate, boreal and subtropical forest ecosystems were different root categories based on root diameter (i.e. fine, medium and large roots), biomass of absorptive and transportive roots and different fine root traits i.e. root tissue density (RTD), specific root length (SRL), and specific root area (SRA) (Prada-Salcedo et al., 2021; Sun et al., 2021). Traditionally, roots with a diameter < 2 mm have been classified as fine roots, characterized as short-lived and primarily involved in absorption. Conversely, roots with a diameter > 2 mm are typically classified as coarse roots, known for their longer lifespan and key role in water and nutrient transport (Finer et al., 2007). Fine roots are vital systems for plants to obtain nutrients and water, and their functions may have an impact on plant growth and survival (Comas et al., 2013; Jagodzinski et al., 2016). Besides the role of nutrient uptake, fine roots in forest trees also influence nutrient availability by depositing C as root exudates (Finzi et al., 2015). Previous studies demonstrated that higher root exudation in the rhizosphere soil of forest due to higher fine root biomass and surface area released more root exudates (Heinzle et al., 2023) (**Figure 3**). Therefore, root-exudate flux appears to be linked to fine-root functional trait coordination across nutrient availability, temperature, and moisture gradients in different forest ecosystems (Nakayama and Tateno, 2018; Sun et al., 2021). Furthermore, previous research suggests that root exudation in forest trees is positively linked to the SRL (Meier et al., 2020). For example, exudation amount of mature *Quercus crispula* plants was augmented fivefold when SRL was doubled (Nakayama and Tateno, 2018). This phenomenon is strengthened by a tissue physiological mechanism, because plentiful metabolites are enriched into the phloem, transported to the root, and then released at the root apical meristem, as size of xylem and phloem is related to high SRL (Farrar et al., 2003) (**Figure 4**). Other research in forest ecosystems has shown that root morphology has a profound impact on root metabolite secretion, as exudation rates were higher when roots were on average thinner and had more root tips (Meier et al., 2015; Zhao et al., 2023). In thinner roots of forest trees, the non-structural C concentration for root formation is lowered, allowing more C to be available for root exudation (Lv et al., 2023).

The variation in root metabolite exudation of trees is influenced by several fine root functional traits, notably including root diameter, RTD, and root nitrogen content. These traits have been observed to account for significant variations in exudate metabolites. For instance, in a forest ecosystem, Williams and de Vries (2020) reported an increase in specific root metabolite exudation with larger root diameters and a decrease with higher RTD. This inverse relationship between metabolite exudation and RTD can be interpreted as an indicator of a conservative trait, which is typically associated with slower growth due to the higher carbon costs involved in structural root development and adaptability to environments with limited nutrient availability. Conservative traits, like high RTD, are usually linked to slower growth rates, greater resilience, and efficiency in resource use, often seen in environments with limited resources. Conversely, acquisitive or competitive traits are associated with faster growth and higher resource turnover, evident in environments with abundant resources. For example, in species like *Poaceae and Quercus*, studies by Guyonnet et al. (2018) and Sun et al. (Sun et al., 2017, 2021) found a positive correlation between root exudation and traits indicative of competitiveness (acquisitive traits) such as root respiration, root tissue nitrogen, SRL, and SRA. On the other hand, a negative correlation was observed with RTD, a conservative trait. This pattern suggests that root exudation tends to align more with acquisitive (competitive) rather than conservative traits. This implies that roots with higher nitrogen content and carbon respiration rates are more likely to release carbon-based root metabolites, while those with higher construction costs, reflecting conservative traits, tend to exude fewer metabolites.

## 5 Metabolite and root traits across soil horizons impact microbial functional groups

Root metabolites contribute plant-derived carbon to the forest’s soil, with the rate of root exudation varying over time, space, and across different environmental conditions (Gao et al., 2021; Keller et al., 2021; Chen et al., 2023). For instances, crucial soil parameters such as organic matter distribution, which varies with soil depth in temperate forest ecosystems, affect both root exudation and microbial composition and function (Schenk, 2005; Tückmantel et al., 2017) (**Figure 1**). Furthermore, a decline in root exudation from topsoil to subsoil is observed due to reductions in soil microbial biomass and nutrient availability, alongside an increase in aromatic carbon in soil organic matter as soil depth increases in a temperate forest ecosystem (Tückmantel et al., 2017). Roots in topsoil provide C-rich substrates that promote microbial breakdown of complex organic compounds (Phillips et al., 2012; Meier et al., 2015, 2017) (**Figure 1**). The allocation of carbon (C) to tree roots and root metabolites can vary under different nutrient regimes at various soil depths, influenced by diverse microbial interactions (**Figure 1**). For instance, ectomycorrhizal fungi enhance the quantity and alter the composition of carbon exuded by host plant’s roots across different soil layers in Norway spruce forest (Rineau and Garbaye, 2009). Furthermore, the decrease in tree root exudation with increasing soil depth is influenced by factors such as decreasing temperature, lower bulk density, and higher oxygen supply and organic matter input in the topsoil compared to the subsoil (Schenk, 2005; Neumann and Römheld, 2007; Rineau and Garbaye, 2009; Jakoby et al., 2020; Proctor and He, 2021) (**Figure 1**). In forest ecosystem, leaf and root litter, as well as root exudation, are principal sources of fresh organic C input to subsoils (Rumpel and Kögel-Knabner, 2011; Mueller et al., 2015; Angst et al., 2016; Rawlik et al., 2022) (**Figure 1**). Therefore, the structure of a forest plant’s root system is crucial in determining the vertical distribution of organic matter and nutrients within the soil (Iversen, 2010; **Figure 1**). The nature of fine roots of forest trees varies across soil depths, with deeper layers typically having primary roots and upper layers populated by finer, fibrous roots (Trocha et al., 2017; Pang et al., 2022) (**Figure 1**). This variation in root types across soil layers could affect root exudation patterns, with higher exudation and fine root turnover in topsoil compared to deep soil (De Graaff et al., 2014; Tückmantel et al., 2017) (**Figure 1**).

Distribution of different root categories (fine or coarse) across soil horizons in forest ecosystem also shape the microbial community composition (Makita et al., 2011; Li et al., 2020; Wang et al., 2020) (**Figure 3**). Fine roots in the topsoil of forest ecosystem attract more diverse and species-rich fungal and bacterial communities (Liu et al., 2022). Interestingly, fine roots, rather than coarse ones, exhibited higher bacterial abundance, suggesting that trees with a higher proportion of fine roots (or root hairs) might attract diverse microbial communities for mutualistic benefits (Pan et al., 2023). For instance, in a subtropical forest, abundance of Proteobacteria, Actinobacteria and Chloroflexi were higher in fine roots with high SRL (Pan et al., 2023). This may be due to the higher surface area of fine roots in the topsoil allowing greater nutrient and metabolite transport compared to coarse roots in forest ecosystem (Wang et al., 2020; Heinzle et al., 2023) (**Figure 3**). In this context of fine root traits, higher overall fungal richness in the topsoil is associated with higher SRL, as observed in the cultivation of single tree species like *Carpinus betulus* and *Quercus robur* (Prada-Salcedo et al., 2021) (**Figure 4**). Conversely, fungal richness is lower in soils associated with trees like *Quercus cerris* and *Quercus ilex*, which supply more resources for higher RTD to support longer root lifespan. Similarly, these root characteristics facilitate connections between symbiotic fungi and fine roots (Fernandez and Kennedy, 2016; Beidler and Pritchard, 2017) (**Figure 4**). The thickness of absorptive fine roots differs by tree species, and fine root diameter significantly impacts symbiotic fungal communities more than other root characteristics (Ma et al., 2018) because cortex segment size is crucial for nutrient acquisition via symbiotic mycorrhizal fungi (Bergmann et al., 2020) (**Figure 4**). SRL is a measure of the relative amount of very fine roots in a root system, and trees with high SRL have thinner roots (Ma et al., 2018), with lower cortex (Kong et al., 2014) and reduced colonization of AM or EM fungi (Brundrett and Tedersoo, 2018) in forest trees. Thus, it can be hypothesized that a reduced root diameter may lead to a smaller cortical area, potentially affecting the colonization by mycorrhizal fungi (**Figure 4**). This hypothesis is supported by the findings of Guo et al. (2008) and Liu et al. (2015), who explored the relationship between root diameter and mycorrhizal colonization in trees of temperate and subtropical forest ecosystems. They noted that a reduced root diameter provides a smaller cortical area, which can limit the environment available for mycorrhizal fungi. Furthermore, root diameter is positively correlated with AM, EM and saprotrophic fungal abundance and diversity but negatively correlated with pathogenic fungal abundance and diversity in different forest ecosystems (Eissenstat et al., 2015; Chen et al., 2018; Dai et al., 2023). This finding is also supported by Semchenko et al. (2018) in a temperate ecosystem, who demonstrated that roots of high SRL were linked with an increased diversity of fungal pathogens and decreased diversity of fungal symbionts, as SRL is inversely related to root diameter (**Figure 4**). Thus, the nature of root exudation and root traits, such as specific root length and diameter, which vary with soil depth, play a crucial role in shaping the composition and diversity of microbial populations, thereby impacting the ecological balance and nutrient cycling within forest ecosystems.

## 6 Influence of climate factors on root traits, root exudation and microbiome

### 6.1 Drought effects

Change in plant traits in forest ecosystem is a crucial strategy by which tree species can survive under adverse environmental conditions (Henn et al., 2018; Pérez-Ramos et al., 2019). It is well recognized that tree leaf and root characteristics exhibit considerable pliability in response to climate change (Bardgett et al., 2014b; Paź-Dyderska et al., 2020; Paź-Dyderska and Jagodziński, 2023) such as variation in sunlight, temperature (Keenan and Niinemets, 2016) and precipitation (Fry et al., 2018). For instances, tree species adapted to arid environments often show increased root-to-shoot ratios, aiding in drought resistance by optimizing water uptake relative to water loss through transpiration (Kozlowski and Pallardy, 2002; Hartmann, 2011). Although drought typically reduces fine root biomass, thus conserving water by reducing transpirational surface area, the stability of certain traits like specific root length (SRL) and root tissue density (RTD) contributes to maintaining water and nutrient uptake efficiency under stress conditions (Arend et al., 2011; Herzog et al., 2014). Furthermore, adjustments in root architecture, such as increased biomass in very fine roots, enhance the plant’s ability to explore soil for moisture, thereby improving drought resilience (Olmo et al., 2014). These trait adaptations collectively enhance the drought resistance of plants, allowing for more efficient water usage and better survival during dry periods. Thus, drought stress significantly alters root traits and metabolite profiles in plants, profoundly influencing the rhizosphere microbiome (**Figure 4**). Drought-induced changes in the rhizosphere microbiome are partly governed by plant-specific responses, especially in root characteristics, including changes in root morphology or shifts in belowground carbon distribution patterns, crucial for forest trees adaptation to drought (Ma et al., 2018; Hildebrand et al., 2023). For example, in a forest ecosystem, metabolomic analyses of the *Populus trichocarpa* plant have shown that drought conditions increase the plant’s investment in carbon (C) and nitrogen (N) metabolisms, as evidenced by the root tissue profiles of amino acids, fatty acids, and phenolic glycosides (Veach et al., 2020) and thus tolerance to drought condition. Notably, several of these metabolites have been found to positively correlate with the alpha-diversity of root-associated fungal and bacterial communities, although such correlations were not observed in soil microbial communities (Veach et al., 2020). However, Timm et al. (2018) found that the bacterial alpha-diversity of *Populus deltoides* rhizosphere was reduced under drought stress and certain core bacterial operational taxonomic units (OTUs) were correlated with specific plant metabolites in the roots, such as amino acids and aromatics. Additionally, the fungal response to drought varies significantly by trophic guild, linked to root morphological adjustments to water scarcity. For instance, in forest ecosystems, specifically regarding *Populus euphratica* under drought conditions, it was observed that the abundance of symbiotrophic microbes was negatively correlated with SRL. Conversely, the abundance of saprotrophic microbes showed a positive correlation with SRL (Xia et al., 2023). This implies that drought conditions influence the relationship between root morphology and the types of microbial communities present in the rhizosphere. However, Lv et al. (2023), observed that drought increased root diameter, tissue density, nitrogen concentration, exudation C, and mycorrhizal colonization, but decreased non-structural C concentration, and specific root length and area in a temperate forest ecosystem which may increase the drought tolerance of plants. While the specific impacts of drought on rhizosphere microbial groups in forest ecosystems, particularly through changes in root morphology and root metabolites, are not fully understood, studies in grasslands and agricultural ecosystems, as detailed in the information presented below, offer relevant insights. For example, under drought conditions, some plants modify their root architecture, often increasing the root:shoot ratio by developing finer roots to enhance water uptake with a lower carbon investment and reduced reliance on symbiotic fungi (Comas et al., 2013). In contrast, other species develop thicker roots and are more dependent on symbiotic relationships for water and nutrient acquisition (Comas et al., 2012; Lozano et al., 2022). This response is coupled with changes in root exudation patterns due to decreased photosynthetic activity, impacting the microbial community in the rhizosphere (Hartman and Tringe, 2019; Chen et al., 2022). The shift in root exudates under drought, including various primary (e.g. organic acids) and secondary metabolites (e.g. terpenoids), creates a different soil microenvironment. This alteration, influenced by plant-specific metabolite exudation, affects the microbial community dynamics, potentially impacting microbial species abundance and diversity (Fitzpatrick et al., 2018; Ochoa-Hueso et al., 2018; Williams and de Vries, 2020). These metabolites exudation change helps plants resilience to drought stress by enhancing beneficial symbiotic interactions. For example, strigolactone and flavonoids excretion plays a crucial role in enhancing plants’ resilience to abiotic stress. For example, there is a notable association between the amount of strigolactones and flavonoids released and the rate of AM fungal colonization in plants under water scarcity conditions (Ruiz-Lozano et al., 2016). Plant root exudates can selectively attract certain bacteria that contribute to enhancing drought resilience. For instance, under drought conditions, plants shown to secrete higher levels of organic acids, notably malic acid, in addition to fumaric, malonic, succinic, and oxalic acids (Henry et al., 2007). Malic acid acts as a potent attractant for the bacterium *Bacillus subtilis*, observed within the soybean rhizosphere (Allard-Massicotte et al., 2016), a microbial chemoattractant known to bolster drought tolerance of plants through the stimulation of osmolyte production (Gagné-Bourque et al., 2016). Additionally, beneficial interactions have been documented between plants and other bacteria such as *Pseudomonas putida* and *Bacillus amyloliquefaciens* through these organic acid exudation, which help mitigate the adverse effects of drought (Kumar et al., 2016). While these studies provide valuable information, further research is needed to understand the specific dynamics of microbial groups and their relationships with root metabolites and morphological traits in forests under drought conditions.

### 6.2 Influence of temperature

Temperature is another environmental factor which influenced root morphology and exudation. It was demonstrated that root metabolite exudation of forest trees increases at higher mean annual temperature (MAT) and atmospheric CO_2_ (Meier et al., 2020) and thus influences rhizosphere microbes. Zadworny et al. (2017) revealed that distinct root morphological characteristics of Scots pine are linked to MAT, with bigger root diameter and lower SRL and RTD with lower MAT in northern Europe. The declining MAT caused an upsurge of structural protection (increased xylem/phloem layer) in fine transport roots of *Pinus sylvestris* (Zadworny et al., 2017) which might constrain the growth of pathogens. By contrast, low MAT enhance the growth of mutualistic and saprotrophic fungi in *Pinus sylvestris*, as larger cortex with high RTD offers more space for fungal colonization (Zadworny et al., 2017). In beech forests, Leuschner et al. (2022) found that root exudate release significantly increased, nearly tripling, as average daily temperatures rose from 10°C to 20°C. This points to a substantial temperature impact on carbon flux from roots to soil. However, the study by Yang et al. (2023) revealed that in an alpine coniferous forest, air temperature had a significant effect on root exudation by altering the characteristics and biomass of fine roots. Their findings suggest that the adaptation of carbon provision by roots and the morphological traits of fine roots to lower temperatures are key factors leading to decreased trees root exudation. Complementing this, Pausch and Kuzyakov (2018) observed that higher temperatures in beech forests stimulate the plant’s demand for nitrogen and other nutrients, leading to increased root exudation to support soil microbes, thereby boosting microbial activity. Furthermore, warming significantly influences tree root exudation and, consequently, the rhizosphere microbiome (Yu et al., 2024). For instance, in a temperate forest, Yu et al. (2024) observed that warming led to a reduction in secondary metabolite exudation, causing a decline in the complexity of soil bacterial and fungal communities in the rhizosphere, unlike in non-rhizosphere soil. Furthermore, long-term warming in temperate forest influenced root exudation (Heinzle et al., 2023) and decreased fine root biomass (Wang et al., 2021c), and influenced the rhizosphere microbial community. Together, these studies underscore the impact of warming on root-soil-microbe interactions, highlighting the importance of both the quantity and diversity of tree root metabolites in maintaining soil microbial diversity and ecosystem functioning under changing climate conditions (Philippot et al., 2013; Venturi and Keel, 2016; Yu et al., 2024).

### 6.3 Effects of elevated CO_2_ and N availability

The response of soil microbes to nitrogen (N) deposition and elevated CO_2_ in forest ecosystems varies, likely influenced by the unique survival strategies of these environments which involve alterations in the root traits and exudates of trees. For example, rising levels of atmospheric CO_2_ are expected to enhance the supply of carbon (C) and nitrogen (N) to fine roots, especially in N-deficient forest ecosystems (Norby and Jackson, 2000). This increase in fine-root distribution affects soil C storage and N cycling since fine roots in forests have a rapid turnover rate (Gill and Jackson, 2000) and contributes significantly to the soil’s C and N contents (Iversen et al., 2008), thereby impacting soil microbial communities. Studies have found that trees in CO_2_-enriched environments increase C allocation to roots via root exudates, providing energy for microbial processes that convert unavailable forms of N into forms that forest plants can use (Langley et al., 2009; Schleppi et al., 2012). These C-rich exudates stimulate free-living microbes to release enzymes that break down N-rich soil organic matter. In addition, under high CO_2_ conditions, trees may directly supply C to mycorrhizal fungi to access N from soil organic matter (Talbot and Treseder, 2010). However, soil N availability might negatively impact root exudation. For instance, in a forest ecosystem, the addition of nitrogen was observed to negatively impact the root exudates of *Pinus tabulaeformis*, leading to an inhibition of fungal and bacterial populations in the rhizosphere soil (Jing et al., 2023). However, it was observed that roots of different diameters responded differently to nitrogen (N) addition and microbial colonization. Specifically, N addition enhanced the concentration of carbon (C) metabolites in roots, with finer roots showing more pronounced changes than thicker roots and increased the rhizosphere microbial diversity and bacterial quantity, but decreased fungal quantity (Jing et al., 2023). Furthermore, Meier et al. (2020) found that at acidic, N-poor sites, the quantity of C released with root exudation is closely positively related to SRL and soil acidity and negatively to fungal abundance and activity in a mature beech forest. Moreover, studies in tropical forest ecosystems showed a positive relationship between the amount of root exudation and the nitrogen content in roots, while there was a negative correlation with the density of root tissues (Sun et al., 2021), which might have some influence on microbial populations. For example, Weng et al. (2013) found that nitrogen (N) application increased the abundance of bacterial in the rhizosphere and this change was correlated to an increase in the production of amino acids and carbohydrates in the root exudates in a mangrove forest. N availability also plays a critical role in shaping the development and structure of the trees fine root system. It has been shown that N availability in a temperate forest leads to changes in root traits, such as increased SRL, reduced root diameter, and higher root biomass allocation (Li et al., 2021), thereby affecting mutualistic or pathogenic fungal communities (**Figure 4**). Furthermore, in temperate forest systems, research on three *Quercus* species revealed a significant link between root exudation and the nitrogen content in root tissues (Ataka et al., 2020), which in turn influenced the woodland microbial community.

Thus, significant changes in root morphological characteristics and metabolomics profiles along drought, temperature, elevated atmospheric CO_2_ and N availability gradients, explain the changes of root microbial communities and adaptive strategies of plants under global climate change scenarios.

## 7 Conclusions

In conclusion, this review summarizes the general changes in the relative abundance and activity of soil microbial communities, alongside alterations in root traits and metabolites across soil horizons, and their associations with climatic drivers of change. We have concluded that different soil horizons, root characteristics, and environmental conditions collectively influence microbial communities in forest ecosystems. It explains that various soil layers, each with distinct physical and chemical properties, harbor specific microbial populations. The diversity in microbial life is largely dependent on the soil’s organic matter content and nutrient availability, with particular emphasis on the distinct distributions of fungi such as ectomycorrhizal and saprotrophic species across different soil horizons. Root exudates, significantly influenced by root morphology including diameter, tissue density, and specific root length, are crucial in shaping the composition and activity of these soil microbial communities. Furthermore, the review underscores the role of environmental factors like drought, temperature, and nutrient availability in altering root traits and exudation patterns. These environmental changes, in turn, affect the structure and function of microbial communities. Stated simply, linking rhizospheric microbial interactions to root morphology and metabolomics may help us to reserve below ground resources for maintenance and enhancement of natural ecosystem services in the context of climate change. Furthermore, this review explains the adaptive strategies of plants by harnessing root trait plasticity and microbial interactions under global climate change scenarios. However, there remain some open questions regarding the environmental effects on plant traits and their implications for the rhizosphere microbiome in forest ecosystems. These questions are particularly pertinent to plant functional traits concerning the functions of root metabolites, changes in root morphology, and their relationships to microbial functional groups.

## Author contributions

PM: Conceptualization, Data curation, Formal analysis, Methodology, Writing – original draft, Writing – review & editing. KH: Investigation, Supervision, Validation, Writing – review & editing. AS: Data curation, Validation, Writing – review & editing. AJ: Resources, Supervision, Writing – review & editing. JA-R: Methodology, Visualization, Writing – review & editing. DM: Methodology, Writing – review & editing. JM: Conceptualization, Investigation, Methodology, Project administration, Supervision, Writing – review & editing.
